# *N*-Benzyl­isatin

**DOI:** 10.1107/S1600536812006575

**Published:** 2012-02-17

**Authors:** M. Schutte, H. G. Visser, A. Roodt, H. Braband

**Affiliations:** a Department of Chemistry, University of the Free State, PO Box 339, Bloemfontein 9300, South Africa; b Institute of Inorganic Chemistry, University of Zurich, Winterthurerestrasse 190, CH-8057 Zurich, Switzerland

## Abstract

In the title compound, C_15_H_11_NO_2_, two C—H⋯O hydrogen bonds are observed in the crystal structure, as well as π–π stacking with a centroid–centroid distance of 3.623 (2) Å. The planarity of the two ring systems is illustrated by very small deviations of all the atoms from these planes [largest deviations = 0.003 (3) and 0.010 (3) Å for the phenyl and fused-benzene rings, respectively]. The dihedral angle between these two planes is 77.65 (9)°.

## Related literature

For literature regarding the biological properties of *N*-benzyl­isatin, see: Palmer *et al.* (1987 ▶); Goldschmidt & Llewellyn (1950 ▶); Wei *et al.* (2004 ▶); Frolova *et al.* (1988 ▶); Akkurt *et al.* (2006 ▶). For background regarding the functionalization of isatin, see: Schutte (2011 ▶). For a similar structure, see: Akkurt *et al.* (2006 ▶).
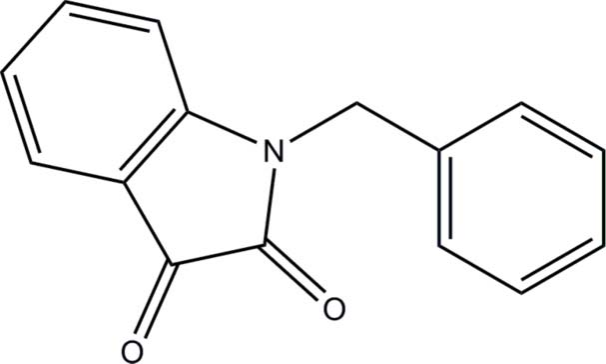


## Experimental

### 

#### Crystal data

C_15_H_11_NO_2_
*M* *_r_* = 237.25
Monoclinic, 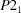

*a* = 6.5766 (5) Å
*b* = 4.8877 (4) Å
*c* = 18.2211 (13) Å
β = 98.316 (7)°
*V* = 579.55 (8) Å^3^
*Z* = 2
Mo *K*α radiation
μ = 0.09 mm^−1^
*T* = 183 K
0.34 × 0.07 × 0.02 mm

#### Data collection

Oxford Xcalibur Ruby CCD diffractometer
Absorption correction: multi-scan (*CrysAlis PRO*; Oxford Diffraction, 2007 ▶) *T*_min_ = 0.881, *T*_max_ = 0.921
5347 measured reflections
2095 independent reflections
1264 reflections with *I* > 2σ(*I*)
*R*_int_ = 0.068

#### Refinement

*R*[*F* ^2^ > 2σ(*F* ^2^)] = 0.062
*wR*(*F* ^2^) = 0.128
*S* = 0.99
2095 reflections
163 parameters
1 restraint
H-atom parameters constrained
Δρ_max_ = 0.15 e Å^−3^
Δρ_min_ = −0.19 e Å^−3^

### 

Data collection: *CrysAlis PRO* (Oxford Diffraction, 2007 ▶); cell refinement: *CrysAlis PRO*; data reduction: *CrysAlis PRO*; program(s) used to solve structure: *SHELXS97* (Sheldrick, 2008 ▶); program(s) used to refine structure: *SHELXL97* (Sheldrick, 2008 ▶); molecular graphics: *DIAMOND* (Brandenburg & Putz, 2005 ▶); software used to prepare material for publication: *WinGX* (Farrugia, 1999 ▶).

## Supplementary Material

Crystal structure: contains datablock(s) global, I. DOI: 10.1107/S1600536812006575/ez2282sup1.cif

Structure factors: contains datablock(s) I. DOI: 10.1107/S1600536812006575/ez2282Isup2.hkl

Supplementary material file. DOI: 10.1107/S1600536812006575/ez2282Isup3.cml

Additional supplementary materials:  crystallographic information; 3D view; checkCIF report

## Figures and Tables

**Table 1:** Hydrogen-bond geometry (Å, °)

*D*—H⋯*A*	*D*—H	H⋯*A*	*D*⋯*A*	*D*—H⋯*A*
C3—H3⋯O2^i^	0.95	2.41	3.213 (5)	143
C9—H9*A*⋯O2^ii^	0.99	2.59	3.525 (4)	159

Symmetry codes: (i) 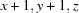; (ii) 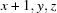.

**Fig. 1.**
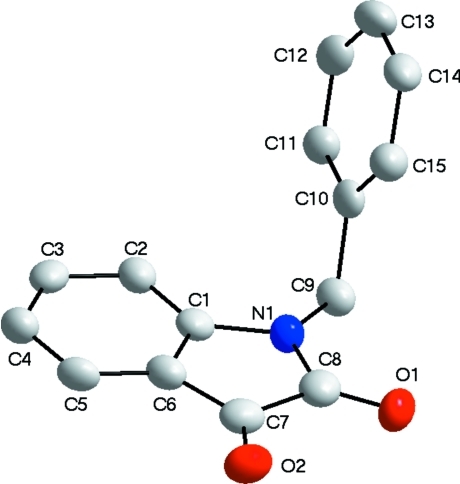
Representation of the title compound, showing the numbering scheme and displacement ellipsoids (50% probability).

**Fig. 2.**
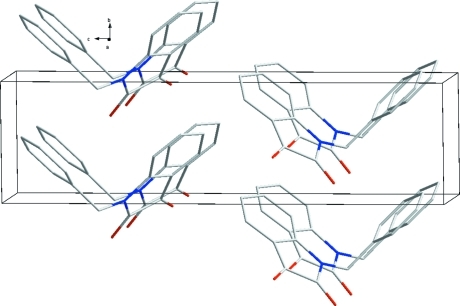
Packing of the title compound in the unit cell.

**Fig. 3.**
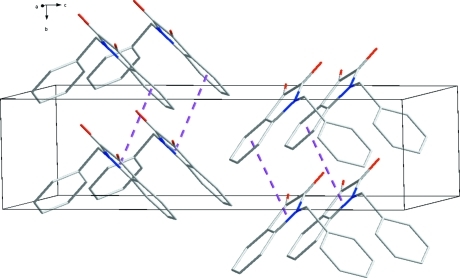
Observed π-π stacking in the crystal structure, indicated by dashed lines (hydrogen atoms omitted for clarity).

## supplementary crystallographic information

### Comment

The molecule isatin has a variety of biological activities. It can cause anxiety but can also be used as a sedative. It can also act as an anticonvulsant agent and can block the binding of an agonist at the atrial natriuretic peptide receptors (Palmer *et al.*, 1987; Goldschmidt & Llewellyn, 1950; Wei *et al.*, 2004; Frolova *et al.*, 1988; Akkurt *et al.*, 2006). Benzylisatin was synthesized to explore the reactivity of the amide group in the isatin molecule and to investigate its possible biological reactivity as free ligand or as bidentate ligand as part of the Re(I) tricarbonyl complex. The coordination of bi- and tridentate ligand systems to the Re(I) tricarbonyl synthon is part of an ongoing study. The amide group was functionalized in the isatin molecule to illustrate the pH dependent keto-enol tautomerisation of the molecule to coordinate in a bidentate fashion to the Re(I) metal centre. By functionalizing the amide, keto-enol tautomerisation is no longer possible and the derivatized isatin cannot coordinate to the Re(I) core (Schutte, 2011).

The title compound, *N*-benzylisatin, crystallized in the monoclinic spacegroup P2_1_ with one molecule in the asymmetric unit. The carbonyl carbon to oxygen distances of 1.221 (5) Å and 1.209 (4) Å compare well with the structure of Akkurt *et al.* (2006) of 1.2061 (18) Å and 1.2091 (17) Å, and the rest of the bond distances and angles of the two structures are also similar. The torsion angles C15—C10—C9—N1 and O1—C8—C7—O2 are 57.0 (5) ° and 0.5 (6) ° for this structure and 53.41 (16) ° and 1.7 (2) ° for the reported structure by Akkurt *et al.* (2006), respectively. The planarity of the two ring systems, C10—C11—C12—C13—C14—C15 and N1—C1—C2—C3—C4—C5—C6—C7—C8, are illustrated by very small deviations of all the atoms from these planes, with the largest deviations 0.003 (3) Å for C13 and 0.010 (3) for C4 respectively. The isatin group in the structure of Akkurt *et al.* is almost planar, with a maximum deviation of 0.058 (1) Å for atom O2. The dihedral angle between the two planes is calculated as 77.65 (9) ° in this structure and 87.08 (5) ° in the structure of Akkurt *et al.*.

The main difference between the structure of *N*-benzylisatin reported here and that by Akkurt *et al.* is the packing as a result of the different space groups, *P*2_1_ and *P*2_1_/c, respectively. In this structure report, the benzylisatin molecules pack in a head-to-toe fashion along the *a* axis and in layers when viewed along the *b* axis (Figure 2). In the structure by Akkurt *et al.* the molecules pack in a staggered head-to-head fashion when viewed along the *c* axis.

Three C—H···O hydrogen bonds are observed in the structure of *N*-benzylisatin. One is an intramolecular hydrogen bond and the other two are intermolecular hydrogen bonds to two neighbouring molecules. Some π-stacking is observed in the crystal structure of *N*-benzylisatin between neighbouring molecules, with a centroid-to-centroid distance of 3.623 (2) Å. This is illustrated in Figure 3.

### Experimental

The preparation was performed under strict Schlenk conditions. Isatin (0.2 g, 1.36 mmol) was dissolved in dry dimethylformamide (3 ml). Powdered calcium hydride (0.191 g, 4.54 mmol) was added to the mixture and stirred at 45 °C for 30 minutes. Benzylchloride (0.258 ml, 2.04 mmol) was added to the mixture and stirred at room temperature for 16 h. The reaction mixture was dried, dissolved in ethylacetate and washed three times with water. The combined ethylacetate layers were dried with Na_2_SO_4_. The product was purified with column chromatography with DCM:Hex 1:1 as eluent and monitored with TLC. The resulting orange product was dried under vacuum. Orange crystals were grown from a methanol solution of the product.

### Refinement

Aromatic H atoms were positioned geometrically and allowed to ride on their parent atoms, with *U*_iso_(H) = 1.2*U*_eq_(parent) of the parent atom with a C—H distance of 0.95. The methene H atoms were placed in geometrically idealized positions and constrained to ride on their parent atoms with *U*_iso_(H) = 1.2*U*_eq_(C) and at a distance of 0.99 Å.

### Crystal data

**Table:**

C_15_H_11_NO_2_	*F*(000) = 248
*M**_r_* = 237.25	*D*_x_ = 1.36 Mg m^−^^3^
Monoclinic, *P*2_1_	Mo *K*α radiation, λ = 0.71073 Å
*a* = 6.5766 (5) Å	Cell parameters from 972 reflections
*b* = 4.8877 (4) Å	θ = 3.1–29.2°
*c* = 18.2211 (13) Å	µ = 0.09 mm^−^^1^
β = 98.316 (7)°	*T* = 183 K
*V* = 579.55 (8) Å^3^	Plate, orange
*Z* = 2	0.34 × 0.07 × 0.02 mm

### Data collection

**Table:**

Oxford Xcalibur Ruby CCD diffractometer	2095 independent reflections
Graphite monochromator	1264 reflections with *I* > 2σ(*I*)
Detector resolution: 10.4498 pixels mm^-1^	*R*_int_ = 0.068
ο oscillation scan	θ_max_ = 25.3°, θ_min_ = 3.1°
Absorption correction: multi-scan (*CrysAlis PRO*; Oxford Diffraction, 2007)	*h* = −7→7
*T*_min_ = 0.881, *T*_max_ = 0.921	*k* = −5→5
5347 measured reflections	*l* = −21→20

### Refinement

**Table:**

Refinement on *F*^2^	Primary atom site location: structure-invariant direct methods
Least-squares matrix: full	Secondary atom site location: difference Fourier map
*R*[*F*^2^ > 2σ(*F*^2^)] = 0.062	Hydrogen site location: inferred from neighbouring sites
*wR*(*F*^2^) = 0.128	H-atom parameters constrained
*S* = 0.99	*w* = 1/[σ^2^(*F*_o_^2^) + (0.0406*P*)^2^] where *P* = (*F*_o_^2^ + 2*F*_c_^2^)/3
2095 reflections	(Δ/σ)_max_ = 0.001
163 parameters	Δρ_max_ = 0.15 e Å^−^^3^
1 restraint	Δρ_min_ = −0.19 e Å^−^^3^

### Special details

**Table:**

Experimental. CrysAlis Pro (Oxford Diffraction Ltd, 2007) Empirical absorption correction using spherical harmonics, implemented in SCALE3 ABSPACK scaling algorithm.
Geometry. All s.u.'s (except the s.u. in the dihedral angle between two l.s. planes) are estimated using the full covariance matrix. The cell s.u.'s are taken into account individually in the estimation of s.u.'s in distances, angles and torsion angles; correlations between s.u.'s in cell parameters are only used when they are defined by crystal symmetry. An approximate (isotropic) treatment of cell s.u.'s is used for estimating s.u.'s involving l.s. planes.
Refinement. Refinement of *F*^2^ against ALL reflections. The weighted *R*-factor *wR* and goodness of fit *S* are based on *F*^2^, conventional *R*-factors *R* are based on *F*, with *F* set to zero for negative *F*^2^. The threshold expression of *F*^2^ > 2σ(*F*^2^) is used only for calculating *R*-factors(gt) *etc*. and is not relevant to the choice of reflections for refinement. *R*-factors based on *F*^2^ are statistically about twice as large as those based on *F*, and *R*- factors based on ALL data will be even larger.

### Fractional atomic coordinates and isotropic or equivalent isotropic displacement parameters (Å^2^)

**Table:**

	*x*	*y*	*z*	*U*_iso_*/*U*_eq_	
C8	0.7610 (6)	−0.1172 (9)	0.7201 (2)	0.0419 (11)	
C7	0.6174 (6)	0.0052 (8)	0.6537 (2)	0.0398 (10)	
C6	0.7413 (5)	0.2130 (8)	0.6238 (2)	0.0356 (10)	
C5	0.6944 (5)	0.3865 (8)	0.5635 (2)	0.0396 (10)	
H5	0.5629	0.3827	0.534	0.048*	
C4	0.8453 (6)	0.5654 (8)	0.5477 (2)	0.0406 (11)	
H4	0.8183	0.6855	0.5065	0.049*	
C3	1.0351 (6)	0.5702 (8)	0.5917 (2)	0.0395 (10)	
H3	1.1357	0.6967	0.5804	0.047*	
C2	1.0833 (6)	0.3952 (8)	0.6520 (2)	0.0371 (10)	
H2	1.2146	0.3986	0.6815	0.045*	
C1	0.9315 (5)	0.2159 (9)	0.6670 (2)	0.0339 (10)	
C9	1.1290 (5)	−0.0424 (8)	0.7759 (2)	0.0395 (10)	
H9A	1.2449	−0.0664	0.7473	0.047*	
H9B	1.1091	−0.2179	0.8011	0.047*	
C10	1.1851 (6)	0.1760 (8)	0.8340 (2)	0.0358 (10)	
C11	1.3769 (6)	0.2963 (9)	0.8421 (2)	0.0456 (11)	
H11	1.4729	0.2444	0.8104	0.055*	
C12	1.4297 (7)	0.4925 (10)	0.8963 (3)	0.0543 (13)	
H12	1.5617	0.575	0.9013	0.065*	
C13	1.2940 (8)	0.5685 (9)	0.9425 (2)	0.0561 (13)	
H13	1.3316	0.7019	0.9799	0.067*	
C14	1.1016 (7)	0.4498 (10)	0.9345 (2)	0.0550 (12)	
H14	1.0056	0.5023	0.9661	0.066*	
C15	1.0489 (6)	0.2540 (8)	0.8801 (2)	0.0451 (11)	
H15	0.9164	0.1727	0.8747	0.054*	
N1	0.9447 (4)	0.0189 (6)	0.72476 (17)	0.0355 (9)	
O1	0.7208 (4)	−0.2996 (6)	0.76148 (16)	0.0584 (9)	
O2	0.4417 (4)	−0.0659 (6)	0.63438 (16)	0.0561 (9)	

### Atomic displacement parameters (Å^2^)

**Table:**

	*U*^11^	*U*^22^	*U*^33^	*U*^12^	*U*^13^	*U*^23^
C8	0.052 (3)	0.036 (3)	0.040 (3)	0.004 (2)	0.013 (2)	−0.007 (2)
C7	0.039 (2)	0.040 (3)	0.042 (3)	0.000 (2)	0.012 (2)	−0.012 (2)
C6	0.038 (2)	0.035 (2)	0.032 (3)	0.006 (2)	−0.0002 (19)	−0.005 (2)
C5	0.034 (2)	0.044 (3)	0.039 (3)	0.001 (2)	−0.0022 (18)	−0.009 (2)
C4	0.051 (3)	0.036 (3)	0.033 (3)	0.000 (2)	0.000 (2)	−0.001 (2)
C3	0.054 (3)	0.028 (2)	0.038 (3)	−0.001 (2)	0.013 (2)	−0.007 (2)
C2	0.046 (2)	0.035 (2)	0.031 (2)	0.004 (2)	0.0050 (18)	−0.003 (2)
C1	0.040 (2)	0.034 (2)	0.029 (2)	0.001 (2)	0.0087 (19)	−0.008 (2)
C9	0.045 (2)	0.038 (2)	0.034 (2)	0.006 (2)	0.0028 (18)	−0.001 (2)
C10	0.045 (2)	0.032 (2)	0.028 (3)	0.006 (2)	−0.0017 (19)	0.007 (2)
C11	0.045 (3)	0.046 (3)	0.043 (3)	0.004 (2)	0.000 (2)	0.003 (2)
C12	0.059 (3)	0.048 (3)	0.050 (3)	−0.002 (2)	−0.011 (2)	0.010 (3)
C13	0.084 (4)	0.044 (3)	0.035 (3)	−0.004 (3)	−0.010 (3)	−0.003 (2)
C14	0.080 (3)	0.046 (3)	0.041 (3)	0.005 (3)	0.014 (2)	−0.004 (3)
C15	0.060 (3)	0.039 (3)	0.037 (3)	−0.005 (2)	0.009 (2)	−0.001 (2)
N1	0.0379 (18)	0.036 (2)	0.032 (2)	−0.0020 (17)	0.0025 (15)	0.0040 (18)
O1	0.071 (2)	0.0487 (19)	0.058 (2)	−0.0078 (18)	0.0194 (16)	0.008 (2)
O2	0.0411 (16)	0.057 (2)	0.071 (2)	−0.0102 (16)	0.0099 (14)	−0.0093 (18)

### Geometric parameters (Å, º)

**Table:**

C8—O1	1.221 (5)	C9—N1	1.449 (4)
C8—N1	1.371 (5)	C9—C10	1.511 (5)
C8—C7	1.544 (5)	C9—H9A	0.99
C7—O2	1.209 (4)	C9—H9B	0.99
C7—C6	1.457 (5)	C10—C15	1.368 (5)
C6—C1	1.379 (5)	C10—C11	1.380 (5)
C6—C5	1.386 (5)	C11—C12	1.384 (6)
C5—C4	1.384 (5)	C11—H11	0.95
C5—H5	0.95	C12—C13	1.364 (6)
C4—C3	1.382 (5)	C12—H12	0.95
C4—H4	0.95	C13—C14	1.380 (6)
C3—C2	1.392 (5)	C13—H13	0.95
C3—H3	0.95	C14—C15	1.385 (6)
C2—C1	1.385 (5)	C14—H14	0.95
C2—H2	0.95	C15—H15	0.95
C1—N1	1.420 (5)		
			
O1—C8—N1	125.7 (4)	C10—C9—H9A	108.8
O1—C8—C7	127.2 (4)	N1—C9—H9B	108.8
N1—C8—C7	107.1 (4)	C10—C9—H9B	108.8
O2—C7—C6	130.8 (4)	H9A—C9—H9B	107.7
O2—C7—C8	124.6 (4)	C15—C10—C11	119.0 (4)
C6—C7—C8	104.6 (3)	C15—C10—C9	120.8 (4)
C1—C6—C5	121.7 (4)	C11—C10—C9	120.2 (4)
C1—C6—C7	107.7 (4)	C10—C11—C12	120.2 (4)
C5—C6—C7	130.6 (4)	C10—C11—H11	119.9
C4—C5—C6	117.9 (4)	C12—C11—H11	119.9
C4—C5—H5	121.1	C13—C12—C11	120.7 (5)
C6—C5—H5	121.1	C13—C12—H12	119.7
C3—C4—C5	120.3 (4)	C11—C12—H12	119.7
C3—C4—H4	119.8	C12—C13—C14	119.4 (4)
C5—C4—H4	119.8	C12—C13—H13	120.3
C4—C3—C2	122.0 (4)	C14—C13—H13	120.3
C4—C3—H3	119	C13—C14—C15	119.8 (4)
C2—C3—H3	119	C13—C14—H14	120.1
C1—C2—C3	117.2 (4)	C15—C14—H14	120.1
C1—C2—H2	121.4	C10—C15—C14	120.9 (4)
C3—C2—H2	121.4	C10—C15—H15	119.6
C6—C1—C2	120.9 (4)	C14—C15—H15	119.6
C6—C1—N1	111.7 (3)	C8—N1—C1	108.9 (3)
C2—C1—N1	127.4 (3)	C8—N1—C9	125.9 (3)
N1—C9—C10	113.6 (3)	C1—N1—C9	124.9 (3)
N1—C9—H9A	108.8		
			
O1—C8—C7—O2	0.5 (6)	N1—C9—C10—C11	−124.2 (4)
N1—C8—C7—O2	−179.1 (4)	C15—C10—C11—C12	0.1 (6)
O1—C8—C7—C6	−179.8 (4)	C9—C10—C11—C12	−178.7 (4)
N1—C8—C7—C6	0.6 (4)	C10—C11—C12—C13	0.3 (6)
O2—C7—C6—C1	179.0 (4)	C11—C12—C13—C14	−0.6 (7)
C8—C7—C6—C1	−0.7 (4)	C12—C13—C14—C15	0.5 (6)
O2—C7—C6—C5	−1.2 (7)	C11—C10—C15—C14	−0.2 (6)
C8—C7—C6—C5	179.1 (4)	C9—C10—C15—C14	178.6 (4)
C1—C6—C5—C4	−0.1 (6)	C13—C14—C15—C10	0.0 (6)
C7—C6—C5—C4	−179.8 (4)	O1—C8—N1—C1	−180.0 (4)
C6—C5—C4—C3	−0.7 (6)	C7—C8—N1—C1	−0.4 (4)
C5—C4—C3—C2	1.1 (6)	O1—C8—N1—C9	5.0 (6)
C4—C3—C2—C1	−0.7 (5)	C7—C8—N1—C9	−175.4 (3)
C5—C6—C1—C2	0.4 (6)	C6—C1—N1—C8	−0.1 (4)
C7—C6—C1—C2	−179.8 (3)	C2—C1—N1—C8	−179.8 (4)
C5—C6—C1—N1	−179.3 (3)	C6—C1—N1—C9	175.0 (3)
C7—C6—C1—N1	0.5 (4)	C2—C1—N1—C9	−4.7 (6)
C3—C2—C1—C6	−0.1 (5)	C10—C9—N1—C8	−111.7 (4)
C3—C2—C1—N1	179.6 (4)	C10—C9—N1—C1	74.0 (4)
N1—C9—C10—C15	57.0 (5)		

### Hydrogen-bond geometry (Å, º)

**Table:**

*D*—H···*A*	*D*—H	H···*A*	*D*···*A*	*D*—H···*A*
C3—H3···O2^i^	0.95	2.41	3.213 (5)	143
C9—H9*A*···O2^ii^	0.99	2.59	3.525 (4)	159
C9—H9*B*···O1	0.99	2.58	2.941 (5)	101

Symmetry codes: (i) *x*+1, *y*+1, *z*; (ii) *x*+1, *y*, *z*.
